# Editorial

**Published:** 1985-07

**Authors:** 


### Editorial

### A NEW FUTURE FOR THE *JOURNAL*

Much has been written in these columns about our financial problems, in particular about the difficulty of attracting advertisers, an inherent problem for a local journal with a small circulation.

About 5 years ago, Dr. Ian McKee, a practising Edinburgh G.P. started a local journal which he called *Edinburgh Medicine*. He achieved a large circulation by distributing it free to all the doctors in the area and was thus able to attract enough advertising to pay for it. He set up a publishing company, Hermiston Publications and repeated his success by starting a similar journal in Glasgow – *Glasgow Medicine*. This was followed by *Scottish Medicine* and then by *Manchester Medicine*, each new venture adding to the distribution he could offer to advertisers. He has now approached this *Journal* with an invitation to join the association. If we will accept *Bristol Medicine* as a second title, go to A4 paper size and publish bimonthly, Hermiston Publications will bear all the costs within certain agreed limits and distribute it to all the doctors in the West of England. We retain our title, the appointment of the Editor remains the prerogative of the Bristol Medico-Chirurgical Society and the contents of the *Journal* are his decision.

On receiving these proposals the Editor went to Edinburgh to meet Dr. McKee and to take soundings, he was favourably impressed and Dr. McKee agreed to come to Bristol to put his proposals to an augmented meeting of the Editorial Committee. The proposals were carefully examined and the committee unanimously decided to recommend their acceptance. This recommendation was later endorsed by the general committee of the Society and will now be put to the full meeting of the Society in October. If accepted the *Journal* in its new form will appear in January 1986.

Hermiston Publications accept that our *Journal* has a tradition and we are not expected to make great changes in style, nevertheless it has been our policy recently to amplify the scientific content with current affairs and general reading of interest to doctors, this policy will be taken further and doctors will be invited to write on their leisure pursuits. The association is to be based on a gentleman's agreement which can be terminated by either party at will and at short notice. It can only succeed if it is mutually satisfactory and sustained by good personal relationship. In the long term we must produce a *Journal* that advertisers believe that doctors will read and that must be a good aim. In the short term the proposed increases in the Society's subscription will not be needed and we can build up some funds so that if the venture fails we can revert to our former status with something in reserve.

The wider circulation of the *Journal* should appeal to authors as well as to advertisers and we could end up with a better *Journal* than before as well as a free one. It is much to be hoped that the Society will find these proposals attractive and will endorse the recommendation of their committee.

